# Overexpression and cosuppression of xylem‐related genes in an early xylem differentiation stage‐specific manner by the AtTED4 promoter

**DOI:** 10.1111/pbi.12784

**Published:** 2017-07-27

**Authors:** Satoshi Endo, Kuninori Iwamoto, Hiroo Fukuda

**Affiliations:** ^1^ Department of Biological Sciences Graduate School of Science The University of Tokyo Tokyo Japan

**Keywords:** secondary cell wall formation, Arabidopsis, xylem‐specific overexpression, cosuppression, woody biomass engineering, lignocellulose

## Abstract

Tissue‐specific overexpression of useful genes, which we can design according to their cause‐and‐effect relationships, often gives valuable gain‐of‐function phenotypes. To develop genetic tools in woody biomass engineering, we produced a collection of Arabidopsis lines that possess chimeric genes of a promoter of an early xylem differentiation stage‐specific gene, Arabidopsis *Tracheary Element Differentiation‐related 4* (*AtTED4*) and late xylem development‐associated genes, many of which are uncharacterized. The AtTED4 promoter directed the expected expression of transgenes in developing vascular tissues from young to mature stage. Of T2 lines examined, 42%, 49% and 9% were judged as lines with the nonrepeat type insertion, the simple repeat type insertion and the other repeat type insertion of transgenes. In 174 T3 lines, overexpression lines were confirmed for 37 genes, whereas only cosuppression lines were produced for eight genes. The AtTED4 promoter activity was high enough to overexpress a wide range of genes over wild‐type expression levels, even though the wild‐type expression is much higher than *AtTED4* expression for several genes. As a typical example, we investigated phenotypes of pAtTED4::At5g60490 plants, in which both overexpression and cosuppression lines were included. Overexpression but not cosuppression lines showed accelerated xylem development, suggesting the positive role of At5g60490 in xylem development. Taken together, this study provides valuable results about behaviours of various genes expressed under an early xylem‐specific promoter and about usefulness of their lines as genetic tools in woody biomass engineering.

## Introduction

Gene overexpression has been widely used to examine gene functions or to improve useful phenotypes. Unlike gene knockout or knockdown technology, overexpression does not always require genome information of target organisms, allowing similar methods to be applied across species. However, we must pay careful attention to cause‐and‐effect relationships in gene overexpression studies. In higher plants, the cauliflower mosaic virus 35S promoter is one of the most frequently selected promoters to induce very high transgene expression in various cell types (Odell *et al*., 1985). Due to the ubiquitous expression pattern, however, it is more or less inevitable to cause pleiotropic effects in any cell types. Conversely, the 35S promoter‐driven overexpression may be no longer stable in certain developmental stages (Labuz *et al*., 2010; Nilsson *et al*., 1996). In addition, undesired epigenetic regulations often affect transgene expression (e.g. Rajeevkumar *et al*., 2015).

Many efforts have been made in developing technologies to utilize woody biomass as renewable materials or energy source for a sustainable future (Isikgor and Becer, 2015; Menon and Rao, 2012). A majority part of woody biomass consists of xylem cells with secondary cell walls (SCWs). We aimed to develop genetic tools to make SCWs valuable in engineering, as well as to understand molecular mechanisms involved in SCW formation. We first use Arabidopsis to build up our methodology. Arabidopsis plants develop woody xylem cells with SCWs in maturing inflorescence stems and hypocotyls, which offers a model for wood formation (Chaffey *et al*., 2002; Lehmann and Hardtke, 2015; Strabala and MacMillan, 2013; Zhang *et al*., 2011). In order to obtain many key factors in SCW formation, we started constructing a large collection of Arabidopsis overexpression lines to identify distinct phenotypes and causal genes. More uncharacterized genes that were expressed in accordance with the late SCW formation were possible candidates (Demura *et al*., 2002; Kondo *et al*., 2015; Kubo *et al*., 2005; Ohashi‐Ito *et al*., 2010; Yamaguchi *et al*., 2011). In previous reports, some xylem overexpression approaches succeeded in efficiently inducing expected phenotypes by the use of the late SCW formation‐specific promoters (e.g. Petersen *et al*., 2012; Ratke *et al*., 2015; Wilkerson *et al*., 2014). Alternatively, we looked for a promoter capable of inducing an early xylem differentiation stage‐specific overexpression. Such promoter might replace SCW construction steps in new order if genes like the late SCW formation‐associated ones were overexpressed.

In this report, we selected a promoter of Arabidopsis *Tracheary Element Differentiation‐related 4* (*AtTED4*) to modify gene expression for 48 different Arabidopsis genes at the very early stage of xylem differentiation. Each modified gene expression level in homozygous T3 lines was compared with the wild‐type gene expression level. T‐DNA repeat status was also investigated. Moreover, one novel overexpression phenotype is shown. The presented results describe how one plant tissue‐specific promoter drove the expression of different genes and thereby affected wild‐type gene expression in a comprehensive scale that has never been reported before.

## Results and discussion

### Selection of AtTED4 promoter to induce gene expression at an early stage of xylem differentiation

**Figure 1:**
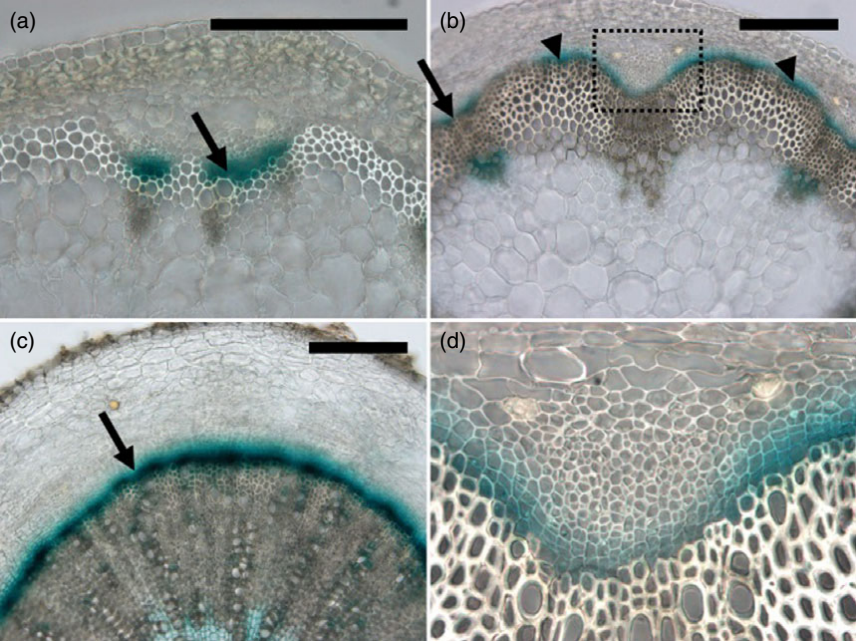
AtTED4 promoter activity. Typical expression patterns of pAtTED4::GUS in developing inflorescence stem (a), maturing stem (b) and hypocotyl (c) in T3 homozygous lines. The boxed area in (b) is magnified in (d). GUS expression was visualized by X‐Gluc staining. Arrows indicate the pAtTED4::GUS signal over a few cell layers of procambium/cambium and immature xylem cells in (a–c). Arrow heads indicate the signal in developing interfascicular fibres in (b). Bars = 200 μm.

35S promoter can induce massive gene expression in not only xylem but also various tissues. However, we required closer cause‐and‐effect relationships in our gene overexpression study. In order to accurately modify gene expression in immature xylem, we looked for an alternative promoter to overexpress genes at an early stage of xylem differentiation. Ze*TED4* was identified as a highly expressed gene in the *Zinnia* tracheary element differentiation system and Ze*TED4* mRNA accumulated in procambium and immature xylem in *Zinnia* plants (Demura and Fukuda, 1994). We examined a promoter activity of its Arabidopsis homologue, At3g18280 (hereafter referred to as *AtTED4*), in Arabidopsis plants to assess whether it reproduced the expression pattern. The pAtTED4::GUS plants showed the fine procambium–immature xylem expression domain in developing vascular tissues from young to mature stage (Figures 1). The GUS expression was also detected in developing interfascicular fibres (Figure 1b). Quantitative PCR analysis of inflorescence stem samples revealed that *AtTED4* mRNA level was 10‐ and 20‐fold higher than that of At5g61480 (*TDR*/*PXY*) and At4g32880 (*ATHB8*), respectively, which were preferentially expressed in procambium and procambium–immature xylem. We selected 48 genes of our interest, many of which are uncharacterized genes specifically expressed in developing xylem cells, for overexpression under the control of the AtTED4 promoter (Table 1).

**Table 1:** Wild‐type expression levels of genes used in this study

Gene	Mean	±SD	Intron	Gene	Mean	±SD	Intron
At5g08480	0.001	0.001	1	At1g27920	1.878	0.114	10
At4g09990	0.002	0.002	1	At3g47400	2.313	0.327	2
At1g10810	0.007	0.003	4	At5g19870	2.635	0.128	0
At5g11540	0.021	0.006	2	At1g09610	2.923	0.180	0
At3g18670	0.034	0.026	3	At1g43790	3.013	0.357	0
At1g59850	0.054	0.008	2	At2g37090	3.950	0.308	2
At3g16920	0.064	0.014	2	At1g58070	3.971	0.086	0
At5g45020	0.084	0.014	5	At5g01730	4.272	0.511	8
At5g54570	0.101	0.028	11	At5g07800	4.323	1.170	6
At1g48280	0.116	0.035	6	At3g59690	4.988	0.323	6
At5g01190	0.142	0.050	5	At1g58370	5.554	0.061	7
At1g29200	0.161	(*n* = 1)	7	At5g40020	6.449	2.092	3
At1g69588	0.181	0.026	0	At2g04780	6.792	0.078	0
At2g20650	0.187	0.009	13	At3g05270	7.264	2.244	3
At5g49900	0.194	0.018	20	At5g17600	7.397	3.301	0
At1g70550	0.225	0.023	5	At1g75410	15.512	2.736	3
At2g38480	0.231	0.021	2	At4g28500	18.555	0.095	2
At5g26330	0.263	(*n* = 2)	1	At5g03170	23.441	3.623	0
At5g08370	0.311	(*n* = 2)	13	At2g38080	27.376	0.503	5
At1g78340	0.386	0.077	1	At4g18780	37.455	2.219	11
At4g08160	0.486	0.189	5	At5g60490	41.971	2.892	0
At4g14940	0.499	0.085	3	At3g16920	107.757	7.235	2
At1g79180	0.756	0.409	2	AtTED4	5.402	2.006	0
At1g31720	0.886	0.045	2	UBQ10	100.000		0
At5g01360	0.997	0.089	4	ATHB8	0.305	0.078	17
At5g59305	1.058	0.145	2	TDR/PXY	0.518	0.096	1

### A survey of multiple T‐DNA insertions at single loci

**Figure 2:**
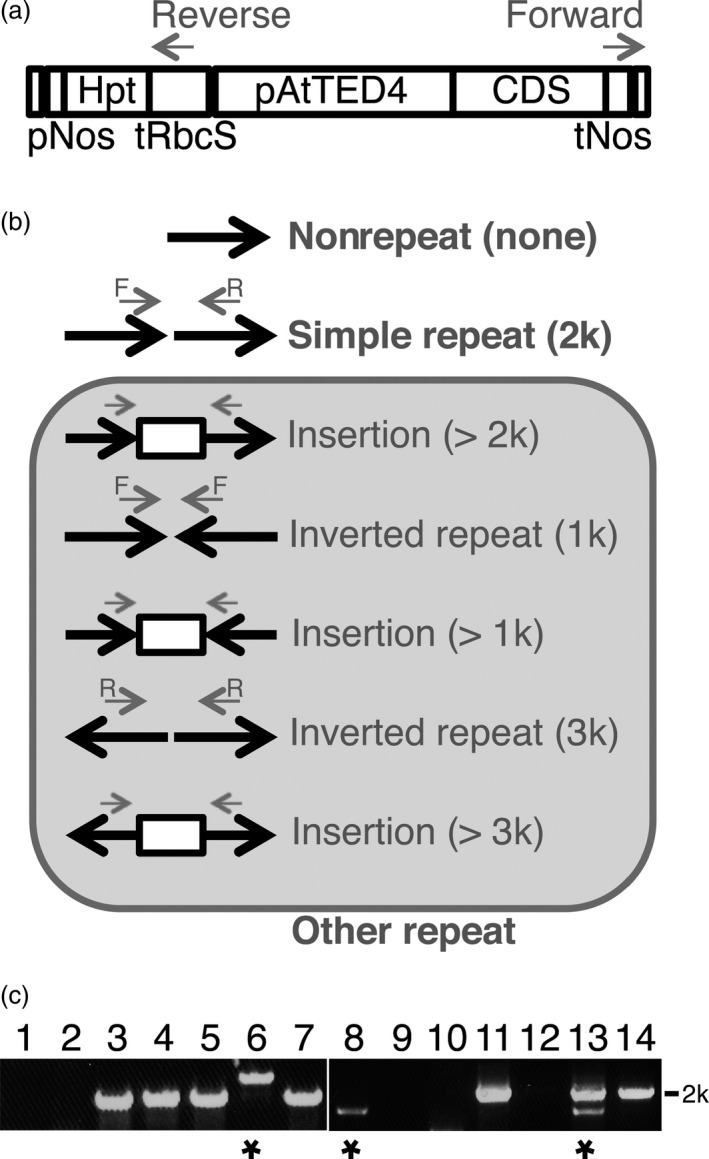
Detection of T‐DNA repeat types. (a) The T‐DNA region used to transform Arabidopsis plants in this study. pNos, the nopaline synthase promoter; Hpt, the hygromycin phosphotransferase‐coding sequence; tRbcS, the Rubisco small subunit terminator; pAtTED4, AtTED4 promoter; CDS, the coding sequence of the selected genes; tNos, the nopaline synthase terminator. Grey arrows indicate the position of the primer set to amplify junction sequences of neighbouring T‐DNAs. (b) A schematic diagram of possible structures of the nonrepeat, the simple repeat or the other repeat of T‐DNAs with PCR products by the indicated primers. Black arrows indicate the direction of T‐DNAs. Grey arrows correspond to the primers in (a). (c) A typical PCR result on gel electrophoresis. Fourteen individual T2 lines were analysed. Lines showing no signal or only the 2 k PCR product were judged as the nonrepeat type or the simple repeat type insertion, respectively. Asterisks mean that those lines have the other repeat type of T‐DNAs.

**Figure 3:**
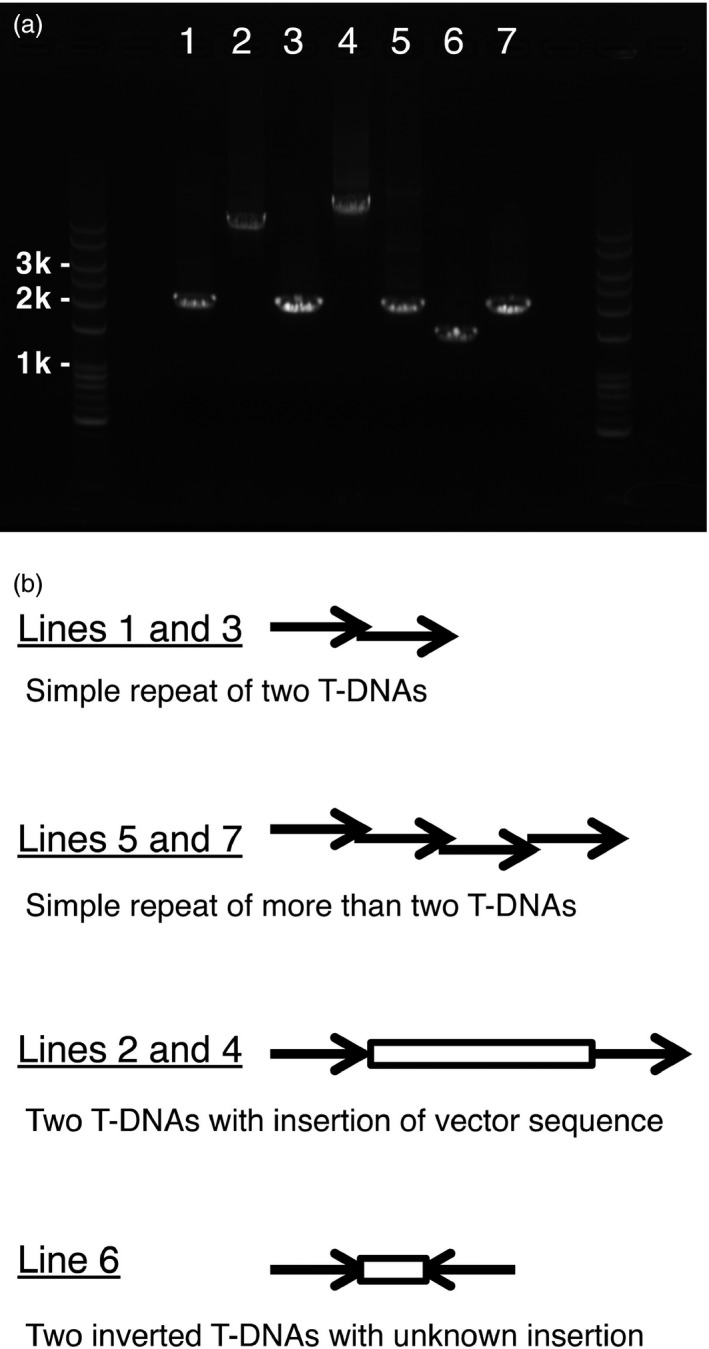
Further investigation of the structure of T‐DNA repeats. (a) PCR products of the junctions of T‐DNA repeats from seven different transgenic lines. Lines showing single PCR products were selected (Lines 1–7). (b) Predicted structures of T‐DNA repeats for each line (see Figure S1).

Transformation of Arabidopsis plants by the floral dip method frequently produces multiple T‐DNA insertions at single loci (De Paepe *et al*., 2009). It has been well documented that such T‐DNA repeats, especially inverted repeats, have often resulted in cosuppression of transgenes and wild‐type genes (e.g. Jorgensen *et al*., 1996). First, we selected transgenic lines in which one or multiple T‐DNA insertion(s) occurred at single loci, based on the 3 : 1 segregation ratio exhibited in the T2 generation. Next, to assess the T‐DNA repeat status of the transgenic lines, we performed PCR‐based observation of junctions of neighbouring T‐DNAs. This simple test was designed to detect whether plants had no repeat or T‐DNA repeats (Figure 2). As a result of total 246 T2 lines for 41 of the 48 genes, 42% and 58% were judged as lines with the nonrepeat type insertion and the repeat type insertion, suggesting that approximately more than a half of transgenic plants have repeats of the transgene (Table 2). Next, we examined the repeat type insertions. We expected that the 2‐kb PCR product results from insertions of directly repeated T‐DNA, the 1‐kb PCR product was from insertions of invertedly repeated T‐DNA, and the other sizes of PCR products may from various structures of insertions (Figure 2b). The 2‐kb PCR products occupied most part of ones from the repeat type insertion (49% of 58%), suggesting that the direct‐repeat type insertion occurs frequently in transgenic plants (Table 2). Therefore, we designed this insertion as the simple repeat. To confirm our expectation, we sequenced the junction of T‐DNA repeats of the 2‐kb PCR products as well as other size of PCR products (Figures 3 and S1). Of four 2‐kb PCR products, two showed a junction (Figure 3, Lines 1 and 3) and two showed multiple junctions (Figure 3, Lines 5 and 7), suggesting that the former two result from a direct repeat of two T‐DNAs and the latter two result from direct repeats of more than two T‐DNAs. Because the 1.5‐kb PCR product was amplified only with the forward primer, we judged that this product results from a structure with an insertion between two inverted T‐DNAs (Figure 3, Line 6). Interestingly, the both PCR products of more than 3 kb showed a direct repeat of two T‐DNAs with a vector sequence insertion between the T‐DNAs (Figure 3, Lines 2 and 4). These results suggested that the repeat type insertion in our transgenic plants contains not only the direct repeat of two T‐DNAs but also of more than two T‐DNAs.

**Table 2:** T‐DNA repeat type

PCR product	T‐DNA repeat type	Numbers of T2 lines
No product	Nonrepeat	103 (42%)
2 k only	Simple repeat	121 (49%)
Other than 2 k	Other repeat	22 (9%)

### Overexpression and cosuppression of xylem‐related genes in an early xylem differentiation stage‐specific manner by the AtTED4 promoter

**Figure 4:**
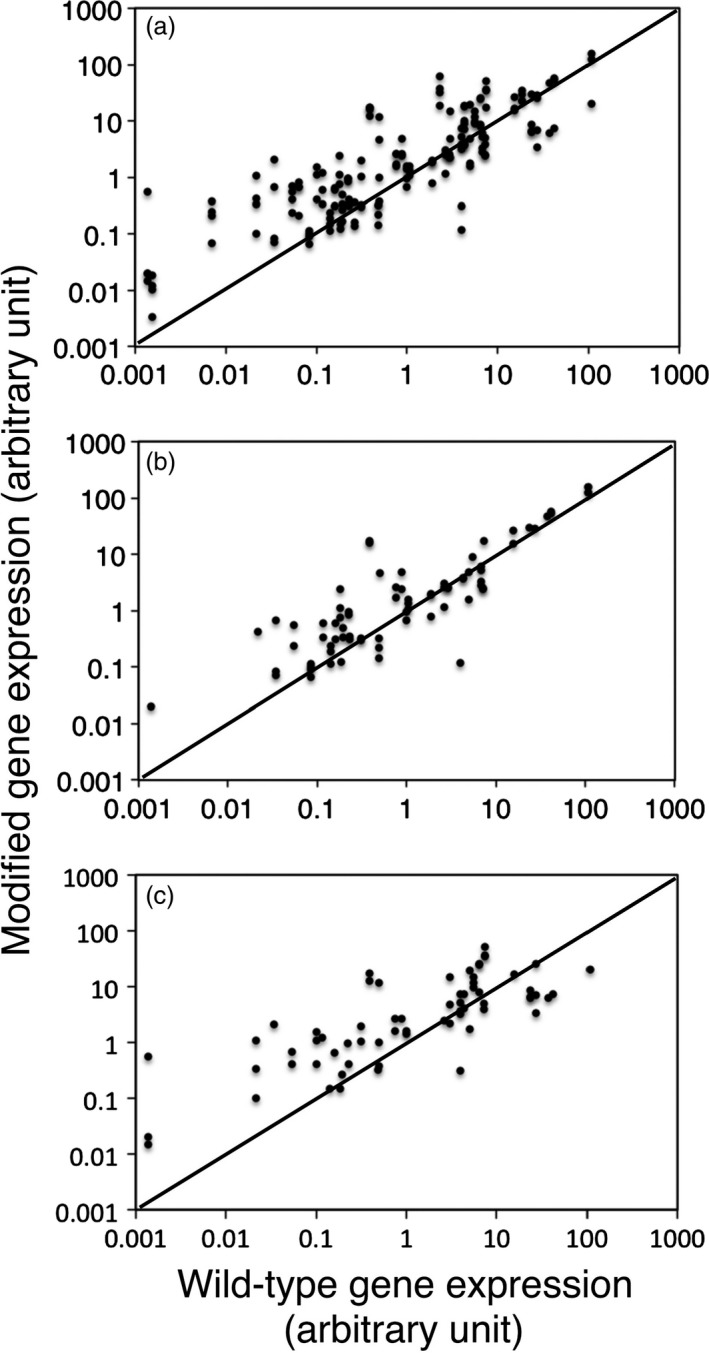
Magnitude of modified gene expression level in individual T3 lines. The graphs plot wild‐type gene expression in *x*‐axis versus modified gene expression in y‐axis for all the examined lines (a), the nonrepeat lines (b) and the simple repeat lines (c). Expression levels were shown by arbitrary unit relative to *UBQ10* level as 100. Diagonal auxiliary lines mean no modification; 174 lines for 48 genes, 75 lines for 37 genes and 66 lines for 35 genes are plotted in (a), (b) and (c), respectively. Note that 32 of the 174 lines lack the T‐DNA junction data set.

Quantitative PCR analysis revealed modified gene expression levels in total 174 T3 lines for 48 genes and the corresponding wild‐type gene expression levels (Figure 4a; Table 1). Overexpression was observed for 37 genes. In addition, one or two cosuppression lines were also detected for eight of the 37 genes (At5g01360, At1g43790, At3g59690, At5g03170, At2g38080, At4g18780, At5g60490, At3g16920). For eight genes such as At2g20650, At4g08160, At1g27920, At5g19870, At1g09610, At1g58070, At2g04780, At3g05270, however, only cosuppression occurred. Based on the T‐DNA junction survey performed in T2 generation, the plots in Figure 4a were further grouped into the nonrepeat type and the simple repeat type (Figure 4b,c). Due to the increased number of T‐DNAs, the simple repeat type structure was more efficient for overexpression of transgenes than the nonrepeat type structure in general, although the simple repeat type tended to cause cosuppression for genes that are highly expressed in wild‐type plants (Figure 4c).

**Figure 5:**
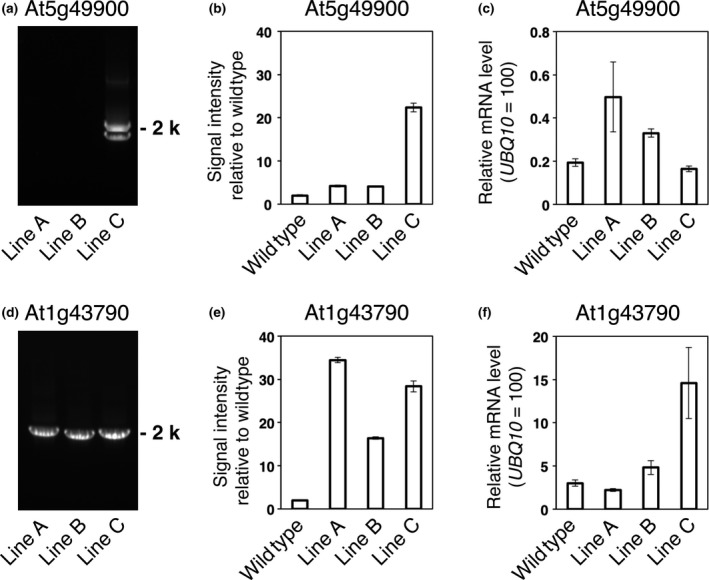
Comparison of T‐DNA structure, the number of T‐DNAs and modified gene expression in At5g49900 and At1g43790 T3 lines. (a–c) Two ‘nonrepeat type’ and one ‘other repeat type’ lines of At5g49900. (d–f) Three ‘simple repeat type’ lines of At1g43790. (a, d) The PCR‐based detection of the junctions as illustrated in Figure 2 for the indicated T3 lines. (b, e) Real‐time PCR‐based estimation of the number of AtTED4 promoter sequence in genomic DNA, when the number of AtTED4 promoter sequence in wild type is assumed to be 2. The data are the means ± SD (*n* = 3). (c, f) Expression level of target genes in wild type and the indicated T3 lines. The data are the means ± SD (*n* = 3).

Next, we selected arbitrarily lines with the nonrepeat type of T‐DNA (At5g49900) and with the simple repeat type of T‐DNA (At1g43790), and examined the relationship between T‐DNA numbers and gene expression levels in the lines (Figure 5). Lines A and B of At5g49900 had the insertion of singly copy of T‐DNA per genome (Figure 5a,b), and their *AtTED4* mRNA level increased about two times more than that in the wild type (Figure 5c). In contrast, Line C of At5g49900 had multiple copies of T‐DNA and its *AtTED4* mRNA level was not more than that of the wild type (Figure 5a–c). Line C of At5g49900 had a 2‐kb and a <2‐kb PCR products (Figure 5a). The <2‐kb product suggests an inverted repeat of T‐DNA, which may result in the repression of *AtTED4* mRNA level. Lines A to C of At1g43790 were simple repeat type lines (Figure 5d), and their copy numbers varied from 7 to more than 15 per genome (Figure 5e). Increases in T‐DNA numbers appeared to associate with overexpression levels in Lines B and C of At1g43790, but Line A, which had the highest copy number, showed the cosuppression of At1g43790 gene expression (Figure 5f). Increases in T‐DNA numbers appeared to associate with overexpression levels on one hand but to result in cosuppression on the other hand. Schubert *et al*. (2004) showed a close relationship between the gene number and the overexpression/cosuppression event using exogenous genes in Arabidopsis. Our study using various endogenous genes is consistent with their result.

The AtTED4 promoter could overexpress At1g75410, At4g28500, At4g18780, At5g60490 and At3g16920 genes in the nonrepeat type lines, even though their wild‐type expression level was much higher than that of wild‐type *AtTED4* expression level (Figure 4b; Table 1). In contrast, the AtTED4 promoter only moderately overexpressed genes whose wild‐type expression level was much lower than that of wild‐type *AtTED4* expression level (Figure 4). These results suggest that an intrinsic AtTED4 promoter activity was high enough to overexpress all the genes analysed in this study, although each transgene expression level in mRNA amount was somehow restricted down towards its wild‐type gene expression level by unknown mechanisms.

Christie *et al*. (2011) reported that endogenous genes avoided gene silencing through intron splicing, and then, small RNA densities were high in exons of intronless genes. Therefore, we compared cosuppression frequency between intronless and intron‐possessing genes. Seven of the nine intronless genes caused cosuppression by their overproduction, while nine of the 36 intron‐possessing genes caused cosuppression (Tables 1 and S3). This result indicated that overexpression of intronless genes tend to cause cosuppression, which was consistent with the previous report (Christie *et al*., 2011), even when a tissue‐specific promoter was used. For cosuppression events, the threshold model has been proposed and carefully examined (Nagaya *et al*., 2005; Schubert *et al*., 2004). They showed that each model reporter gene had its expression threshold for silencing. The threshold probably depends on a quality control machinery of aberrant RNAs for each gene (Hayashi *et al*., 2012; Zhang *et al*., 2015). As we have not found any common features in sequence or gene expression level among the eight genes for which no overexpression lines were obtained, the genes may have their own low threshold level for silencing.

**Figure 6:**
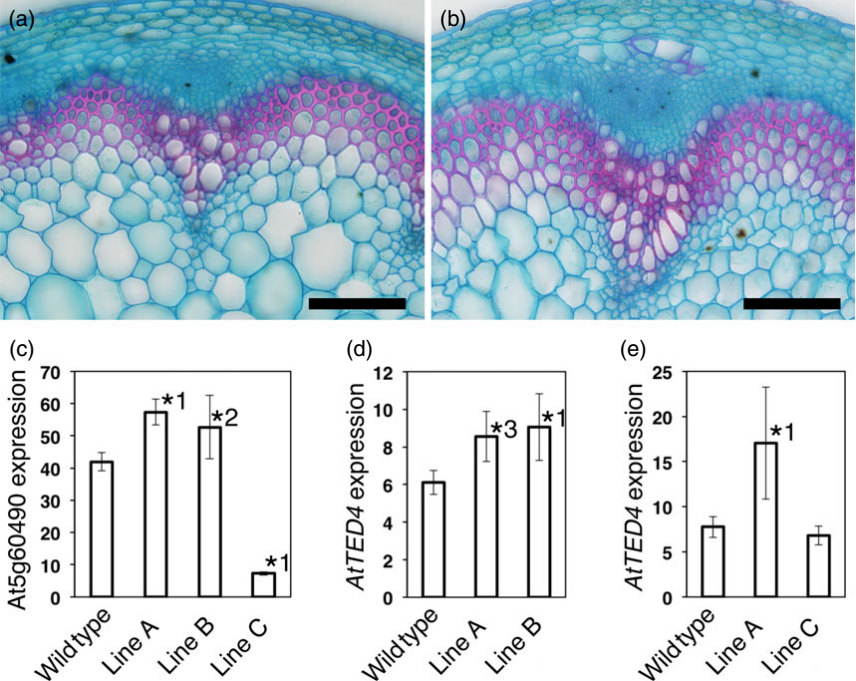
Effect of At5g60490 overexpression on vascular development. (a, b) Developing inflorescence stems of wild‐type (a) and of At5g60490 overexpression plants (b). A typical result observed in T1 lines is shown. The result was confirmed in another At5g60490 overexpression T4 line (Figure S2). The sections were stained with Alcian blue and Safranin. Bars = 100 μm. (c–e) Expression level of At5g60490 (c) and *AtTED4* (d, e) in wild type and three independent At5g60490 T3 lines. Quantitative PCR analyses were performed using Lines A–C in (c), Lines A and B in (d) and Lines A and C in (e), independently. All the data are the means ± SD (*n* = 3). Asterisks indicate the differences between the wild type and each line (*^1^ *P* < 0.05, *^2^ *P* = 0.052, *^3^ *P* = 0.056 in Dunnett's test).

Then, we examined whether the overexpression of such a gene affects xylem formation. To test it, we selected At5g60490 lines, because we obtained both overexpression and cosuppression lines for At5g60490 (Figure 6c). The gene is referred to as *Fasciclin‐like arabinogalactan protein 12* (*FLA12*). *FLA12*‐overexpressing plants displayed increased xylem and interfascicular fibre development (Figures 6 and S2). In order to confirm the overexpression phenotype, we used *AtTED4* this time as a xylem differentiation marker. As expected, *FLA12* overexpression in Lines A and B resulted in an increase in *AtTED4* expression (Figure 6c,d). In Line C, the *FLA12* cosuppression line, however, *AtTED4* expression was not higher than wild‐type level (Figure 6c,e). These results strongly suggest that *FLA12* overexpression accelerated xylem and interfascicular fibre development. FLAs form a subfamily of arabinogalactan proteins, for which a variety of functions have been proposed (Seifert and Roberts, 2007). MacMillan *et al*. (2010) reported a reduction in cellulose content and inflorescence stem strength in *fla11 fla12* double mutant. Recently, *Eucalyptus FLA2*, a closest homologue of Arabidopsis *FLA12*, was shown to be able to alter cellulose deposition in woody tissues in the induced somatic sector analysis when overexpressed by the cauliflower mosaic virus 35S promoter (MacMillan *et al*., 2015). Therefore, *FLA12* is supposed to be a key regulator in SCW formation, although its precise function still remains unknown. In our experiments, immature xylem‐specific overexpression of *FLA12*, which is originally expressed in SCW‐forming cells (MacMillan *et al*., 2010; Ohashi‐Ito *et al*., 2010), caused increased xylem and interfascicular fibre development. There have been few cell wall proteins that are proven to promote xylem differentiation. We reported that xylogen, an arabinogalactan protein with the glycosylphosphatidylinositol anchor, which promotes xylem differentiation through cell–cell interaction (Motose *et al*., 2004). FLAs are also arabinogalactan proteins with Fasciclin domain that is related to cell adhesion domain. Therefore, *FLA12* may function in promoting xylem and interfascicular fibre differentiation through cell–cell interaction.

In conclusion, we found that an early xylem differentiation stage‐specific promoter sequence, pAtTED4, was able to overexpress a variety of genes with different wild‐type expression levels in a range of 0.001–100 arbitrary units (Tables 1 and S4). The AtTED4 promoter‐directed expression of late xylem development‐associated genes was able to induce xylem‐related phenotypes, as typically shown by overexpressing At5g60490. Thus, our comprehensive analysis can be a guide for producing tissue‐specific overexpression lines in a large scale and also provides a collection of lines with AtTED4 promoter chimeric genes, which is useful as genetic tools in woody biomass engineering. Efforts are underway to analyse phenotypes of these lines.

## Experimental procedures

### Plant growth conditions

*Arabidopsis thaliana* (L.) Heynh. accession Columbia (Col‐0) was used as the background for all the lines analysed in this study. Selection of transgenic plants was performed on a 1/2 MS‐based conventional medium containing 15 mg/L Hygromycin B under 10‐h fluorescent white light at 22˚C and 14‐h dark at 20˚C (100 μmol/m^2^/s). Seedlings were further grown on pots with a mixture of vermiculite (VS kakou) and PRO‐MIX BX (Premier Horticulture) under 16‐h LED light at 22 ˚C and 8‐h dark at 20 ˚C (120 μmol/m^2^/s).

### Vector construction and Arabidopsis transformation

The primers used in the following steps are shown in Table S1. The 2.8 kb of 5′ sequence from the translation start site of *AtTED4* was integrated into the *Hin*d III‐ and *Xba* I‐treated pH35GS by In‐Fusion HD Cloning kit (Clontech), resulting in introducing pAtTED4 in place of p35S in pH35GS (Kubo *et al*., 2005). Coding sequences were cloned into the modified destination vector by the Gateway system (Invitrogen). Using GV3101 (pMP90), Arabidopsis plants were transformed with the above‐mentioned constructs by the floral dip method (Clough and Bent, 1998).

### Histology

Fresh GUS‐expressing samples were once treated with 90% acetone and then stained in a GUS detecting solution (2 mm X‐Gluc, 0.5 mm potassium ferricyanide 0.5 mm potassium ferrocyanide, 0.1 m sodium phosphate buffer, pH 7.4). The samples were transferred in 70% ethanol and sectioned at 80 μm thickness.

For Alcian blue/Safranin staining, samples more than 1.1 mm diameter were obtained from basal parts of inflorescence stems more than 20 cm in height, fixed with FAA solution (3.7% formaldehyde, 0.5% acetic acid, 50% ethanol), washed with 70% ethanol and sectioned at 80 μm thickness. The sections were stained with 0.05% Alcian blue for 1 min, followed by 0.005% Safranin for 30 sec, and then washed.

### PCR conditions

Neighbouring T‐DNA junctions were amplified with the following forward and reverse primers: 5′‐AAT CCT GTT GCC GGT CTT GCG AT‐3′ and 5′‐GTT CCA GAA TAA TCA ACG CTG AAT AT‐3′. Template DNAs were simply prepared from immature leaves of young seedlings using an extraction buffer (0.5% SDS, 25 mm EDTA, 250 mm NaCl, 200 mm Tris‐HCl buffer, pH 7.5). A standard PCR for 40 cycles was performed using Ex‐Taq DNA polymerase (Takara).

Quantitative PCR was performed using TaqMan probe system with LightCycler 480 (Roche). Primers and probes used in the analysis are shown in Table S2. Total RNAs were prepared from 5‐ to 10‐cm segments from the top of inflorescence stems of each four plants more than 20 cm in height, using RNeasy Plant Mini Kit (Qiagen). The RNAs were converted to cDNAs by SuperScript III (Invitrogen). Calculated by the threshold cycles and the amplification efficiencies of *UBQ10* and each gene in the same PCR, expression levels were shown by arbitrary unit relative to *UBQ10* level as 100. All the data were analysed in triplicates, except as indicated (Tables 1 and S3).

## Supporting information


**Figure S1** Further investigation of the structure of T‐DNA repeats.

**Figure S2** Effect of At5g60490 overexpression on vascular development.

**Table S1** Primers used for cloning.
**Table S2** Primers and probes used for real‐time PCR.
**Table S3** Magnitude of modified gene expression level in individual T3 lines.
**Table S4** Gene annotation.
