# Supplementary figures and images for: Overexpression and cosuppression of xylem‐related genes in an early xylem differentiation stage‐specific manner by the AtTED4 promoter
Source: Plant Biotechnol J. 2017 Jul 27;16(2):451–8. doi: 10.1111/pbi.12784 (PMC5787829; doi:10.1111/pbi.12784)

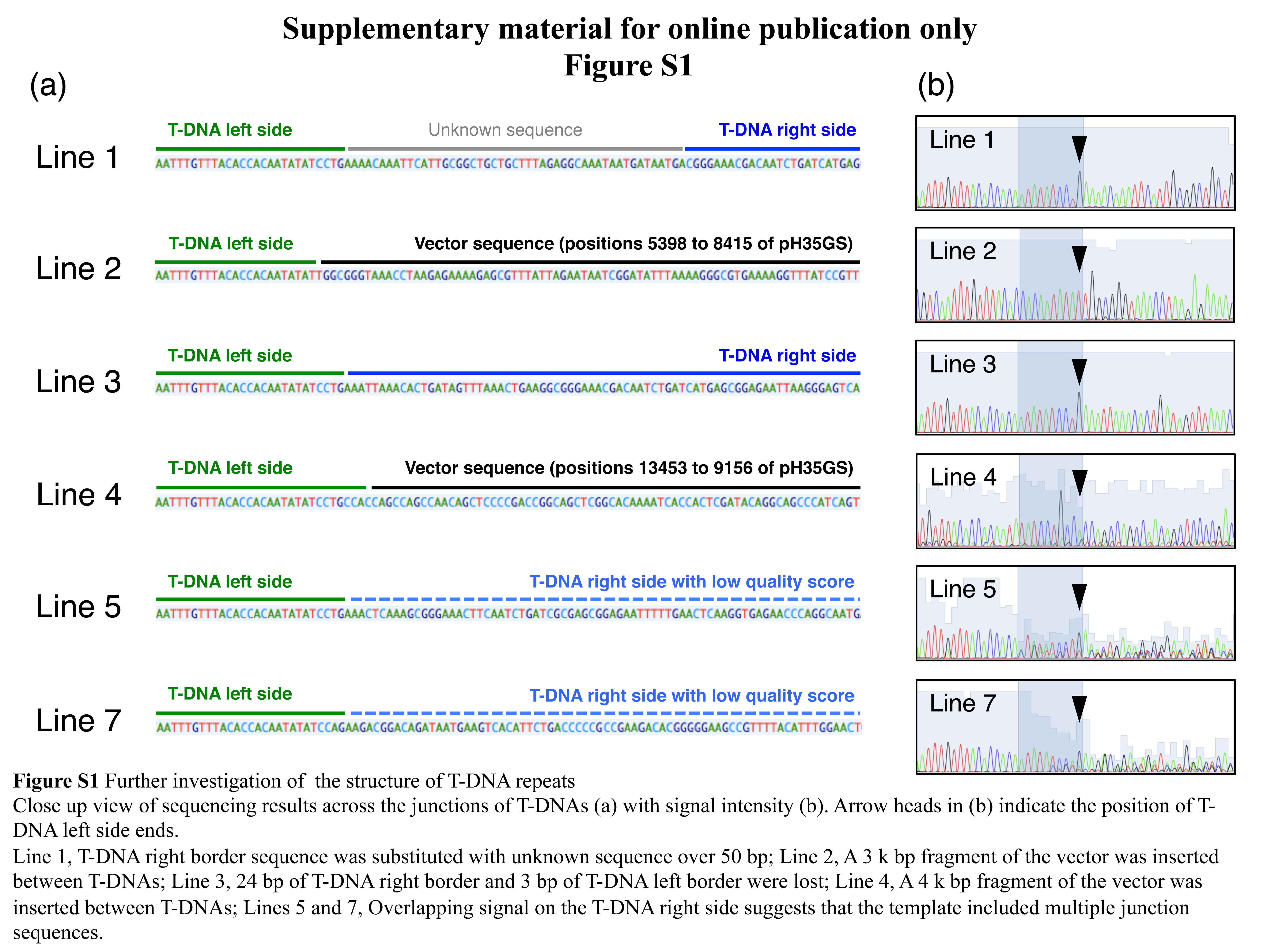

Supplement: Supplementary file 1 — Figure S1 Further investigation of the structure of T‐DNA repeats. [file PBI-16-451-s002.tif]

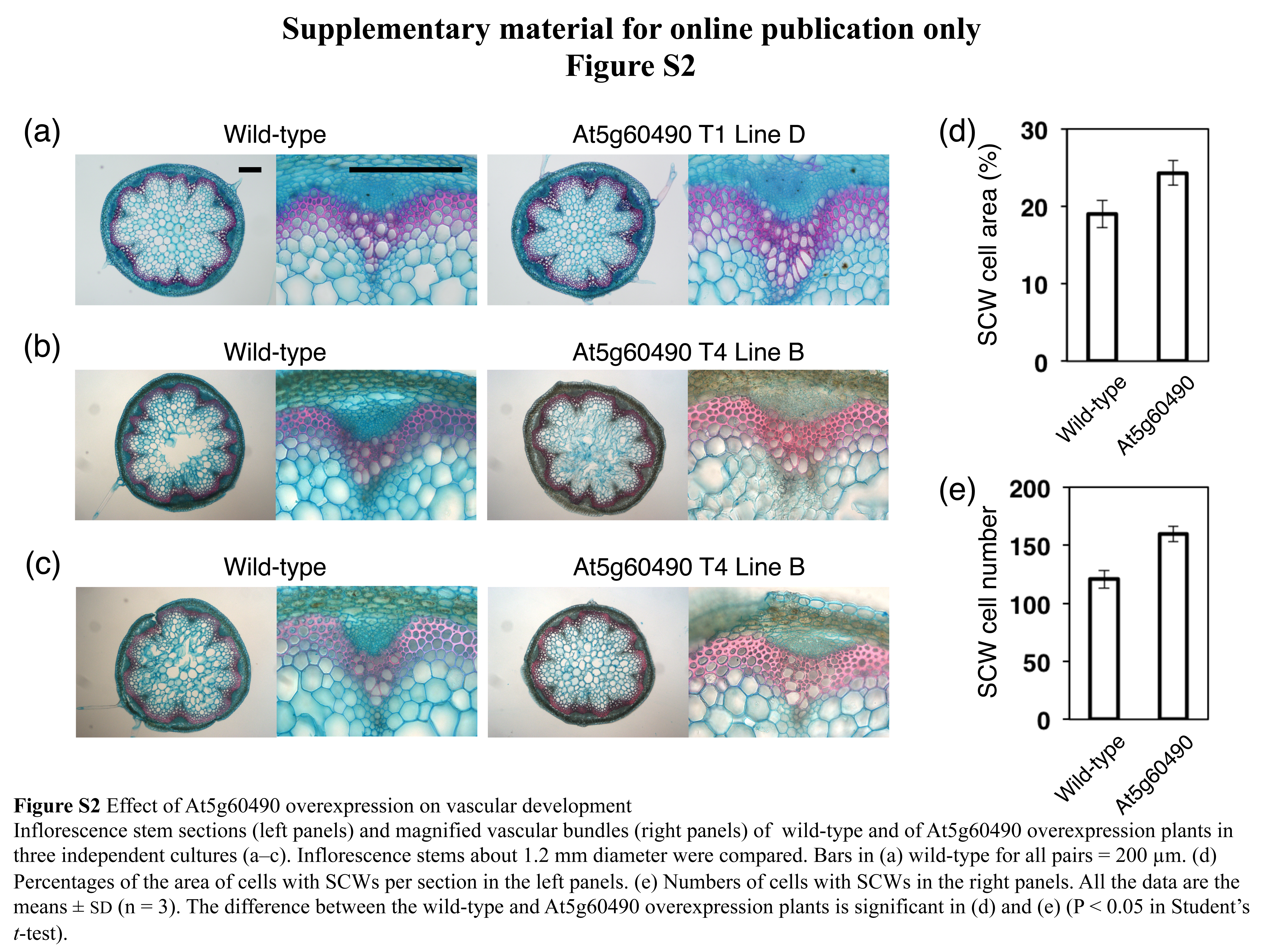

Supplement: Supplementary file 2 — Figure S2 Effect of At5g60490 overexpression on vascular development. [file PBI-16-451-s003.tif]
