# Supplementary material for: Overexpression and cosuppression of xylem‐related genes in an early xylem differentiation stage‐specific manner by the AtTED4 promoter
Source: Plant Biotechnol J. 2017 Jul 27;16(2):451–8. doi: 10.1111/pbi.12784 (PMC5787829; doi:10.1111/pbi.12784)
Supplement: Supplementary file 3 — Table S1 Primers used for cloning. Table S2 Primers and probes used for real‐time PCR. Table S3 Magnitude of modified gene expression level in individual T3 lines. Table S4 Gene annotation. [file PBI-16-451-s001.docx]

**Supplementary material for online publication only**

**Table S1** Primers used for cloning

| **Gene** | **Forward primer** | **Reverse primer** |
| --- | --- | --- |
| At5g08480 | **caccatgaatagcaaagggagtcaaaacgttg** | **ttatggtttgccactcgaattggga** |
| At4g09990 | **caccatgaggaataaatcccaatcattcatcagct** | **tcaaaagcggcgactgatatcc** |
| At1g10810 | **caccatggcagctgcaagtggagt** | **ttatgaagtccaagaagacagtggtg** |
| At5g11540 | **caccatgcgttactctcatactctccag** | **ttacatcactataacttgagctggcga** |
| At3g18670 | **caccatggataccgagaaaggttatccca** | **tcaaagcttaaacactcgccgag** |
| At1g59850 | **caccatgccttccgttcaaatccgttctt** | **tcagttctgtcgacaggcgttt** |
| At3g16920 | **caccatgagtcttcttctacatctctttgctct** | **tcacaatgtcacgctcggaaact** |
| At5g45020 | **caccatggctaggtctggggttgat** | **tcacgaggagaatctgtcgc** |
| At5g54570 | **caccatggaatctttaatgagactcgtcttagttct** | **tcatttctcttggaatttgtactcttgttgttgtt** |
| At1g48280 | **caccatgtcccgcatttccaccac** | **ctacgacagatttccggccatatt** |
| At5g01190 | **caccatggtatttccaattcggattttggttcttttc** | **tcaacatttgggaagatcacttggag** |
| At1g29200 | **caccatgtcaattaagaatggagcagtagatgctt** | **tcaaaatttacacatacactcaggacatctc** |
| At1g69588 | **caccatgttgggttccagtacaagatcaa** | **ttaagaaaatggctgagctttgttgt** |
| At2g20650 | **caccatgaataatctggggaattttggggtttg** | **ttaagctggaggaagtgggc** |
| At5g49900 | **caccatgtttgaggaaaagatcatggatattggtgaa** | **ctatgacatcattctcctgcaagtatagt** |
| At1g70550 | **caccatgtgtttaataggtttgttgggagatgtgt** | **tcatggacatctagggttttttcctg** |
| At2g38480 | **caccatgacgaatcccgataacatgaaacc** | **tcagataaaagaatgagtggagagtttgtaac** |
| At5g26330 | **caccatggcagccattatagtggcg** | **ttaagaagcaaaatttgtaaaggccaaagagacta** |
| At5g08370 | **caccatggttcttcttagtttctccttaagattcattg** | **ttatgccttgcgtcttgtaagagtatacatttt** |
| At1g78340 | **caccatggcggatgaagtgatacttttggattttt** | **ttagacacagtatatcttcctaatcttataggcaaa** |
| At4g08160 | **caccatggagaagaatacaaatacgaaccatacaagt** | **tcacaaagaagagagatcaatggagataacaa** |
| At4g14940 | **caccatgaacacatcaatacttgccatactttttctcata** | **ttacccaatcaaaggatcgttatcgaagaaattt** |
| At1g79180 | **caccatggggaagggaagagcac** | **tcaatgtatcatgagctcgtagttcttcaa** |
| At1g31720 | **caccatggaaattcaaaaacaagataaccgggatg** | **ctacacattttgagtgtgacgtttgtcat** |
| At5g01360 | **caccatgagcttcttgattcctaatagaggagta** | **ttacaaatgagccaataggatccgattc** |
| At5g59305 | **caccatgagaagacatgatatcatcatcaagc** | **tcagtgtttgaccggtgggtgacgatttcc** |
| At1g27920 | **caccatgggttcgcttcagacacc** | **ttatgcctcactctttctgctttctctt** |
| At3g47400 | **caccatgcttagaggcatctttcacatttgc** | **tcacaaccctgacttgaaaggca** |
| At5g19870 | **caccatgggaacattggtgggtcacatttta** | **ttaatcctttgttcgaagctgagagtattcaatttt** |
| At1g09610 | **caccatgaggcctaaagcaaatcaaaaccacaa** | **ctatggacaaaagggtctatttgattctgatt** |
| At1g43790 | **caccatggcctccacggattcagttta** | **tcacgaacgggaaacgactgat** |
| At2g37090 | **caccatgggatctctagagagatcaaagaagaaa** | **tcaggtgcttaaacgtgttcttgtg** |
| At1g58070 | **caccatggatatagaagagatggaaggaaataatcaagt** | **ttaaaagtgcagggtggaagaggta** |
| At5g01730 | **caccatggcattgacgagataccagatac** | **tcactcgctccagctatctgaat** |
| At5g07800 | **caccatggttacgttcacatcagaagcatcaa** | **tcaagaattgaagtttgtgaaatgagagctttgaa** |
| At3g59690 | **caccatggggaagaaaggaagttggttttct** | **tcacgcaaatctgttaaaagccctc** |
| At1g58370 | **caccatggaggtttcaagaaaagacaatgaagag** | **tcacaaaccttgaagatcaacagtaataacct** |
| At5g40020 | **caccatgaaattccagtctacctccttgttacaa** | **ttacgtggaactatggcccca** |
| At2g04780 | **caccatggcaaagatgcaattatcaatctttatcgct** | **tcacaagaacaatgccaccaaacc** |
| At3g05270 | **caccatggaccggaggagctg** | **tcaagattcaatcaagaaatcttcaaatgtagctagt** |
| At5g17600 | **caccatgtcgacaaaccctaacccatg** | **ctaaaggggcagacggtaatttc** |
| At1g75410 | **caccatggctgtgtattaccctaatagtgtc** | **ttagacaacaaagtcgtgtaattgatggg** |
| At4g28500 | **caccatgacttggtgcaatgaccgtag** | **ttaagggataaaaggttgagagtcatgaac** |
| At5g03170 | **caccatggctacttcaagaacattcattttctctaatct** | **ttatatccacagagaagaagaagcagcaat** |
| At2g38080 | **caccatggggtctcatatggtttggtttctattt** | **ttagcacttgggaagatccttagg** |
| At4g18780 | **caccatgatggagtctaggtctcccat** | **ttagcaatcgatcaaaagacagttcagagaaa** |
| At5g60490 | **caccatggaacattctctcatcatcctcct** | **tcacaaataaaaccatgcgagcattacact** |
| At3g16920 | **caccatggtcacattgatagacgaagaaaaagacaaaaaaaaat** | **tcaagaagaggaaccagaactcg** |
| pAtTED4 | tatatgtttgaagctttgattattggggatttcatttgcag | cggtacctattctaggtttcttcttgatcttttgtttgtttc |

**Table S2** Primers and probes used for real-time PCR

| **Gene** | **Forward primer** | **Reverse primer** | **Probe** |
| --- | --- | --- | --- |
| At5g08480 | gcaatgtatgaggccaaagc | tgtcgtaccagtgggcttaaa | #82 |
| At4g09990 | cgtctttggcttaggacacg | tcaatccaagcttcatcttcc | #105 |
| At1g10810 | cgagttgctccttggtcag | caaatttggttgccaattcc | #7 |
| At5g11540 | ttttcttttatgaaacgactgcaa | cgtcgagaagaaaatctctaaacc | #48 |
| At3g18670 | cagtgatcttgatccttgtgatg | tgaatcagatccaaagcaataca | #137 |
| At1g59850 | tggtagaagaagaattagctccact | cccaatgtacgattcattgtctc | #108 |
| At3g16920 | tcaagggatgaaggaagtcg | ctgttgcaaccccgtacc | #142 |
| At5g45020 | aacatcgtcgtcaacagcaa | cggaatgttgatgctgttctt | #50 |
| At5g54570 | tcagccgtgattacaagctc | cagcttgaccctgctctttc | #25 |
| At1g48280 | tgtggattggcttgataaagaac | ctcaggccacttgaaatgct | #106 |
| At5g01190 | cacatagagaagaagtcatcatactcg | tgcttcatttactaccgtttcg | #31 |
| At1g29200 | tggaggccttaatcaacagc | caccaaattggctaggatcttt | #132 |
| At1g69588 | cattcaaatcaagcaagagacg | ggctgagctttgttgtggat | #153 |
| At2g20650 | gagatggagaagcactgcaa | aacgatctttgtccccattg | #44 |
| At5g49900 | ggaacccaaagcttgacaaa | ggtatgtagacttgtctcctttcaga | #127 |
| At1g70550 | acaaggacaaaaaccattgga | caagactcaccggataaactcc | #33 |
| At2g38480 | tcgcattcattgctttagctc | aagaatgagtggagagtttgtaacc | #108 |
| At5g26330 | aaccatagccaacgtagactacaa | ttgtgggttgtattcgaacaag | #165 |
| At5g08370 | gatcaagcaatgagtcgaatgt | aaaatgattccagctgttcca | #91 |
| At1g78340 | ttcgtggacaccaagctg | tgcttctatatactctttcttggctgt | #55 |
| At4g08160 | catgctaaaaggaatcgtcca | gactccgaaaccaggattctc | #63 |
| At4g14940 | cgagattaatgttccaggacaa | tggccaccattctaaccact | #60 |
| At1g79180 | gatctgaagcgtggcaactt | gaagcgattttcgaccactt | #69 |
| At1g31720 | tgtataattcttctgttgttttcttgg | agccaccctttgccgtat | #108 |
| At5g01360 | ctcttatacactgaaagaatcagcttg | tcggacaagactttagcttgaa | #143 |
| At5g59305 | catcatcaagcttctccttctca | gttcggttttgcgatgatct | #143 |
| At1g27920 | tgcggatatttgctcagaga | tttctccggtaagcctctacac | #15 |
| At3g47400 | gcatctttcacatttgcctct | atgtacggcggaggagaac | #143 |
| At5g19870 | tcatcgatccctcatcaactt | gtgtggtctgacgagtggaa | #144 |
| At1g09610 | gatgcaccgactggctactac | ccgcagtgtaaattgctgtc | #92 |
| At1g43790 | cgaagacgcgatcaagaga | aacgggaaacgactgatgat | #22 |
| At2g37090 | gatcaattcccaaagccaag | tgtttctccttcagcttctcg | #24 |
| At1g58070 | ctctttgtttggattttgagtcc | attgttacagtagagggggaacac | #156 |
| At5g01730 | gagatcatacggagcaagtcg | tgaaagtttggtctccctgag | #54 |
| At5g07800 | ggtgcctgatccatttcgta | tccattgatatgtcttgtccactc | #77 |
| At3g59690 | agcagctaagcaacaacaacc | tccattccttagtttctttccaa | #126 |
| At1g58370 | catttgaaaagacaatgtgacaaga | ccaccgggaagctatttctta | #50 |
| At5g40020 | cccaactattacagttatgcctacg | cttggcggagcaagtgac | #63 |
| At2g04780 | gtactgatgtccctccaatgc | tccgaaggagcactaacgat | #15 |
| At3g05270 | tggcgtgagatttgaggtc | ctcctccggtccatatcca | #119 |
| At5g17600 | tcttcctcgacgacgactct | atgagagcgatgaggagagg | #25 |
| At1g75410 | ttgagcatttccttcatcca | atttgcaacctggtttttcg | #112 |
| At4g28500 | cccttcttgtggccataactt | gccttcaagatgctccaaga | #4 |
| At5g03170 | tcgttatagccactacttatggtca | cggttatgttcgttggacct | #1 |
| At2g38080 | aatgagaaagtcatcgttctaggtg | ccagacttaagcgcctcatta | #31 |
| At4g18780 | tttgcctcttgttgcttactgt | cagcatgcttgctaggtttg | #11 |
| At5g60490 | cgatggacattttcctctcaa | acgccggacgtgatatttac | #59 |
| At3g16920 | tcaagggatgaaggaagtcg | ctgttgcaaccccgtacc | #142 |
| AtTED4 | cttctccaccatcggcttt | accacataagcatggcctct | #161 |
| UBQ10 | gaagttcaatgtttcgtttcatgt | ggattatacaaggccccaaaa | #119 |
| ATHB8 | ctcaagagatttcacaacctaacg | tcactgcttcgttgaatcctt | #60 |
| TDR/PXY | attcaaaccgacgaatccat | ttctggtgcaatgtaaccgta | #141 |
| pAtTED4 | gccaacccatcaatgacata | ggttacttggattcgaaatatcatc | #63 |

**Table S3** Magnitude of modified gene expression level in individual T3 lines

|  |  | Line A |  |  | Line B |  |  | Line C |  |  | Line D |  |  |
| --- | --- | --- | --- | --- | --- | --- | --- | --- | --- | --- | --- | --- | --- |
| Gene | Type | Mean | ±SD | Type | Mean | ±SD | Type | Mean | ±SD | Type | Mean | ±SD |  |
| At5g08480 | N | 0.020 | 0.001 | S | 0.015 | 0.001 | S | 0.547 | 0.006 | S | 0.020 | 0.002 |  |
| At4g09990 | U | 0.003 | 0.002 | U | 0.012 | 0.005 | U | 0.018 | 0.011 | U | 0.010 | 0.001 |  |
| At1g10810 | U | 0.068 | 0.014 | U | 0.242 | 0.034 | U | 0.383 | 0.095 | U | 0.208 | 0.043 |  |
| At5g11540 | S | 0.099 | 0.008 | S | 0.332 | 0.048 | S | 1.073 | 0.063 | N | 0.419 | 0.033 |  |
| At3g18670 | N | 0.083 | 0.024 | S | 2.096 | 0.213 | N | 0.682 | 0.020 | N | 0.072 | 0.026 |  |
| At1g59850 | S | 0.401 | 0.022 | N | 0.552 | 0.043 | N | 0.236 | 0.031 | S | 0.685 | 0.076 |  |
| At3g07320 | U | 0.824 | 0.046 | U | 0.210 | 0.210 | U | 0.662 | 0.176 | U | 0.670 | 0.153 |  |
| At5g45020 | N | 0.066 | 0.011 | N | 0.099 | 0.006 | N | 0.090 | 0.010 | N | 0.113 | 0.012 |  |
| At5g54570 | S | 0.405 | 0.051 | S | 1.097 | 0.077 | S | 1.512 | 0.096 |  |  |  |  |
| At1g48280 | N | 0.599 | 0.111 | N | 0.339 | 0.086 | S | 1.193 | 0.061 |  |  |  |  |
| At5g01190 | N | 0.233 | 0.039 | S | 0.147 | 0.053 | N | 0.112 | 0.062 | N | 0.184 | 0.028 |  |
| At1g29200 | U | 0.311 | 0.072 | N | 0.312 | 0.045 | S | 0.650 | 0.066 | N | 0.603 | 0.021 |  |
| At1g69588 | N | 1.119 | 0.237 | N | 0.764 | 0.130 | N | 2.420 | 0.119 |  |  |  |  |
| At2g20650 | N | 0.121 | 0.006 | S | 0.148 | 0.003 |  |  |  |  |  |  |  |
| At5g49900 | N | 0.497 | 0.161 | N | 0.330 | 0.019 | O | 0.165 | 0.013 | S | 0.263 | 0.017 |  |
| At1g70550 | N | 0.939 | 0.065 | N | 0.851 | 0.063 | S | 0.941 | n = 2 |  |  |  |  |
| At2g38480 | S | 0.402 | 0.006 | N | 0.349 | 0.006 | N | 0.306 | 0.009 |  |  |  |  |
| At5g26330 | U | 0.163 | 0.019 | U | 0.367 | 0.039 | U | 0.137 | 0.014 |  |  |  |  |
| At5g08370 | S | 1.948 | 0.114 | N | 0.327 | n = 2 | N | 0.302 | 0.013 | S | 1.035 | 0.431 |  |
| At1g78340 | S | 17.299 | 1.525 | N | 15.906 | 0.747 | N | 17.548 | 2.180 | S | 12.371 | 1.674 |  |
| At4g08160 | N | 0.320 | 0.031 | S | 0.318 | 0.016 | N | 0.223 | 0.071 | N | 0.140 | 0.094 |  |
| At4g14940 | S | 1.003 | 0.148 | S | 11.879 | 0.979 | N | 4.708 | 0.278 | S | 0.382 | 0.041 |  |
| At1g79180 | S | 1.574 | 0.210 | N | 1.699 | 0.097 | N | 2.549 | 0.419 | S | 2.642 | 0.269 |  |
| At1g31720 | S | 2.635 | 0.190 | N | 2.389 | 0.251 | N | 4.842 | 0.173 |  |  |  |  |
| At5g01360 | S | 1.584 | 0.058 | S | 1.418 | 0.111 | N | 0.668 | 0.303 | N | 0.966 | 0.034 |  |
| At5g59305 | N | 1.333 | 0.048 | N | 1.078 | 0.059 | N | 1.545 | 0.063 |  |  |  |  |
| At1g27920 | U | 1.836 | 0.262 | N | 1.953 | 0.326 | N | 0.793 | 0.051 | N | 1.923 | 0.171 | |
| At3g47400 | U | 18.556 | 2.363 | U | 61.314 | 0.660 | U | 31.969 | 2.095 | U | 37.216 | 8.097 | |
| At5g19870 | S | 2.481 | 0.668 | N | 3.041 | 0.297 | N | 2.579 | 0.990 | N | 1.150 | 0.254 | |
| At1g09610 | N | 2.535 | 0.109 | N | 2.616 | 0.200 | U | 2.194 | 0.149 |  |  |  | |
| At1g43790 | S | 2.213 | 0.156 | S | 4.822 | 0.811 | S | 14.604 | 4.115 |  |  |  | |
| At2g37090 | S | 5.194 | 2.660 | U | 3.785 | 1.605 | S | 7.395 | 1.883 |  |  |  | |
| At1g58070 | S | 0.310 | 0.087 | N | 0.119 | 0.015 | S | 3.163 | 0.359 | S | 3.591 | 0.337 | |
| At5g01730 | U | 10.056 | 2.025 | U | 18.883 | 2.155 | U | 8.980 | 2.080 | U | 18.026 | 14.821 | |
| At5g07800 | N | 3.698 | 0.109 | S | 7.156 | 0.149 | N | 3.867 | 0.378 | S | 4.094 | 0.499 | |
| At3g59690 | S | 19.425 | 0.286 | N | 1.586 | 0.281 | N | 4.756 | 0.114 | S | 1.736 | 0.098 | |
| At1g58370 | S | 9.699 | 0.465 | S | 14.750 | 0.510 | N | 9.099 | 0.518 | S | 11.637 | 0.317 | |
| At5g40020 | S | 24.211 | 4.788 | S | 25.199 | 4.019 | U | 8.682 | 1.303 | S | 7.897 | 0.174 | |
| At2g04780 | N | 5.203 | 0.038 | N | 3.239 | 0.203 | N | 6.056 | 0.124 | N | 2.827 | 0.170 | |
| At3g05270 | N | 2.437 | 0.717 | N | 2.519 | 1.076 | S | 3.880 | 2.310 | S | 4.961 | 2.937 | |
| At5g17600 | S | 50.577 | 8.689 | S | 36.501 | 12.945 | N | 17.417 | 1.936 | S | 34.342 | 5.243 | |
| At1g75410 | N | 15.122 | 3.049 | S | 16.865 | 2.802 | N | 26.228 | 1.090 |  |  |  | |
| At4g28500 | U | 29.788 | 4.674 | U | 22.749 | 1.677 | U | 34.251 | 2.002 | U | 22.693 | 1.144 | |
| At5g03170 | N | 29.473 | 3.471 | S | 6.529 | 0.861 | S | 6.266 | 0.855 | S | 8.679 | 0.528 | |
| At2g38080 | S | 25.767 | 1.626 | N | 28.856 | 0.138 | S | 3.357 | 0.098 | S | 6.915 | 0.780 | |
| At4g18780 | N | 46.752 | 1.143 | S | 6.175 | 0.672 |  |  |  |  |  |  | |
| At5g60490 | N | 57.398 | 4.060 | N | 52.715 | 9.833 | S | 7.291 | 0.486 |  |  |  | |
| At3g16920 | S | 20.138 | 1.137 | N | 153.770 | 23.695 | N | 122.426 | 4.861 |  |  |  | |

Type, T-DNA repeat type; N, non-repeat; S, simple repeat; O, other; U, uninvestigated.

Means with underline indicate that values were different from those of wild-type levels (P < 0.05 or P ≈ 0.05 in Dunnett’s test).

**Table S4** Gene annotation

| **Gene** | **Annotation** | **Gene** | **Annotation** |
| --- | --- | --- | --- |
| At5g08480 | VQ motif-containing protein | At5g01360 | TRICHOME BIREFRINGENCE-LIKE 3 |
| At4g09990 | GLUCURONOXYLAN METHYLTRANSFERASE 2 | At5g59305 | unknown protein |
| At1g10810 | NAD(P)-linked oxidoreductase superfamily protein | At1g27920 | microtubule-associated protein 65-8 |
| At5g11540 | L-GULONO-1,4-LACTONE (L-GULL) OXIDASE 3 | At3g47400 | invertase/pectin methylesterase inhibitor superfamily |
| At3g18670 | Ankyrin repeat family protein | At5g19870 | unknown function (DUF716) |
| At1g59850 | ARM repeat superfamily protein | At1g09610 | GLUCURONOXYLAN METHYLTRANSFERASE 3 |
| At3g07320 | O-Glycosyl hydrolases family 17 protein | At1g43790 | TRACHEARY ELEMENT DIFFERENTIATION-RELATED 6 |
| At5g45020 | Glutathione S-transferase family protein | At2g37090 | IRREGULAR XYLEM 9 |
| At5g54570 | BETA GLUCOSIDASE 41 | At1g58070 | unknown protein |
| At1g48280 | hydroxyproline-rich glycoprotein family protein | At5g01730 | SCAR FAMILY PROTEIN 4 |
| At5g01190 | LACCASE 10 | At5g07800 | Flavin-binding monooxygenase family protein |
| At1g29200 | O-fucosyltransferase family protein | At3g59690 | IQ-DOMAIN 13 |
| At1g69588 | CLAVATA3/ESR-RELATED 45 | At1g58370 | ARABIDOPSIS THALIANA XYLANASE 1 |
| At2g20650 | FLYING SAUCER 2 | At5g40020 | Pathogenesis-related thaumatin superfamily protein |
| At5g49900 | GBA2 type family protein | At2g04780 | FASCICLIN-LIKE ARABINOGALACTAN 7 |
| At1g70550 | Protein of Unknown Function (DUF239) | At3g05270 | VESCICLE TETHERING 1 |
| At2g38480 | CASP-LIKE PROTEIN 4B1 | At5g17600 | RING/U-box superfamily protein |
| At5g26330 | Cupredoxin superfamily protein | At1g75410 | BEL1-LIKE HOMEODOMAIN 3 |
| At5g08370 | ALPHA-GALACTOSIDASE 2 | At4g28500 | SCW-ASSOCIATED NAC DOMAIN PROTEIN 2 |
| At1g78340 | GLUTATHIONE S-TRANSFERASE TAU 22 | At5g03170 | FASCICLIN-LIKE ARABINOGALACTAN 11 |
| At4g08160 | putative glycosyl hydrolase family 10 protein | At2g38080 | LACCASE 4 |
| At4g14940 | AMINE OXIDASE 1 | At4g18780 | CELLULOSE SYNTHASE 8 |
| At1g79180 | MYB DOMAIN PROTEIN 63 | At5g60490 | FASCICLIN-LIKE ARABINOGALACTAN 12 |
| At1g31720 | MODIFYING WALL LIGNIN-1 | At3g16920 | CHITINASE-LIKE PROTEIN 2 |
